# Confirmation of Single-Locus Sex Determination and Female Heterogamety in Willow Based on Linkage Analysis

**DOI:** 10.1371/journal.pone.0147671

**Published:** 2016-02-01

**Authors:** Yingnan Chen, Tiantian Wang, Lecheng Fang, Xiaoping Li, Tongming Yin

**Affiliations:** 1 Co-Innovation Center for Sustainable Forestry in Southern China, College of Forestry, Nanjing Forestry University, Nanjing, Jiangsu, China; 2 Forestry Research Institute of Jingdezhen, Jiangxi, China; Pennsylvania State University, UNITED STATES

## Abstract

In this study, we constructed high-density genetic maps of *Salix suchowensis* and mapped the gender locus with an F_1_ pedigree. Genetic maps were separately constructed for the maternal and paternal parents by using amplified fragment length polymorphism (AFLP) markers and the pseudo-testcross strategy. The maternal map consisted of 20 linkage groups that spanned a genetic distance of 2333.3 cM; whereas the paternal map contained 21 linkage groups that covered 2260 cM. Based on the established genetic maps, it was found that the gender of willow was determined by a single locus on linkage group LG_03, and the female was the heterogametic gender. Aligned with mapped SSR markers, linkage group LG_03 was found to be associated with chromosome XV in willow. It is noteworthy that marker density in the vicinity of the gender locus was significantly higher than that expected by chance alone, which indicates severe recombination suppression around the gender locus. In conclusion, this study confirmed the findings on the single-locus sex determination and female heterogamety in willow. It also provided additional evidence that validated the previous studies, which found that different autosomes evolved into sex chromosomes between the sister genera of *Salix* (willow) and *Populus* (poplar).

## Introduction

Dioecism accompanied by sex chromosome dimorphism is common in animals but less prevalent in plants [1]. Only about 4–6% of higher plants show full dioecism [2, 3]. Several dioecious flowering plant species appear to have the XY [4–7] or ZW [8–11] sex determination system without evidence of cytological heteromorphism, which indicates that their sex chromosomes are probably of relatively recent origin. Dioecious plants provide a desirable system to study the genetics and evolution of sex chromosomes [12]. Sex chromosomes are thought to originate from a pair of autosomes, and the sex-determination systems in dioecious plants almost certainly evolve independently from ancestral hermaphrodites that lack sex chromosomes [13–18]. However, compared with the relatively clear sex determination in animals, sex determination in dioecious plants is still poorly characterized.

*Salicaceae* is a family of dioecious woody plants [19], which comprise the sister genera of *Salix* and *Populus*. Thus far, no morphologically different sex chromosomes have been observed in any *Salicaceae* species based on cytological studies [20–22]. In poplars, multiple lines of evidence suggested that chromosome XIX was in the process of evolving into an incipient sex chromosome [10, 23], and studies showed that it was possible that both ZZ/ZW (female heterogamety) and XX/XY (male heterogamety) gender-determining systems could be present in some members of the genus *Populus* [23]. As the sister genera of poplars, gender determination in willow is also drawing much interest. To explain the expression of gender in willow, Alström-Rapaport et al. [24] proposed a multi-locus sex determination mechanism in which the presence of sex chromosomes was unlikely. However, recent molecular studies presented evidence that a single locus governed the sex determination in different willow species [11, 25, 26]; these mapping studies consistently revealed that the female willow was the heterogametic gender, while the male was homogametic, suggesting a ZW sex determination system in willows.

Because the reported sex-determining systems in members of the family *Salicaceae* varies among different studies [10, 27–30], it is desirable to obtain additional evidence to elucidate the gender determination in this family of highly diverged woody plants. Construction of a high-density genetic map is a vital prerequisite for positioning genes underlying traits of interest, which is also critical to gain insight into the sex determination in plants [31, 32]. Linkage maps have been constructed for several willow species [11, 26, 33–38], but only a few of the established maps have been used to study the genetics of willow sex determination [11, 26]. In this study, we established a full-sib mapping pedigree of *S*. *suchowenesis*. We aimed to (1) build high-density genetics maps for the mapping parents; (2) map the gender locus on the established map, and (3) compare our gender mapping result with those of the previous studies.

## Materials and Methods

### Plant material and DNA preparation

The mapping pedigree was created by crossing a diploid female with a diploid male of *S*. *suchowensis* in 2012. The maternal and paternal parents were collected from Nanjing, Jiangsu Province of China, and Linyi, Shandong Province of China, respectively; the permissions were granted by local governments. A pedigree composed of 1,435 progeny was maintained at the Chenwei Forest Farm in Sihong County, Jiangsu Province, China (118°23′N, 33°46′E). A subset of 92 progeny was randomly selected for field trial, genetic map construction and mapping the gender locus. The field studies did not involve any endangered or protected species, and the administrative office of Chenwei Forest Farm authorized the sample collection.

Total genomic DNA was extracted from fresh leaf tissue following the standard cetyltrimethylammonium bromide method (CTAB) with minor modifications [39]. DNA concentration was measured on a Nanodrop 2000 (Thermo Scientific, MA, USA), and DNA quality was assessed by 1% agarose gel electrophoresis.

### Marker generation and map construction

**Fig 1:**
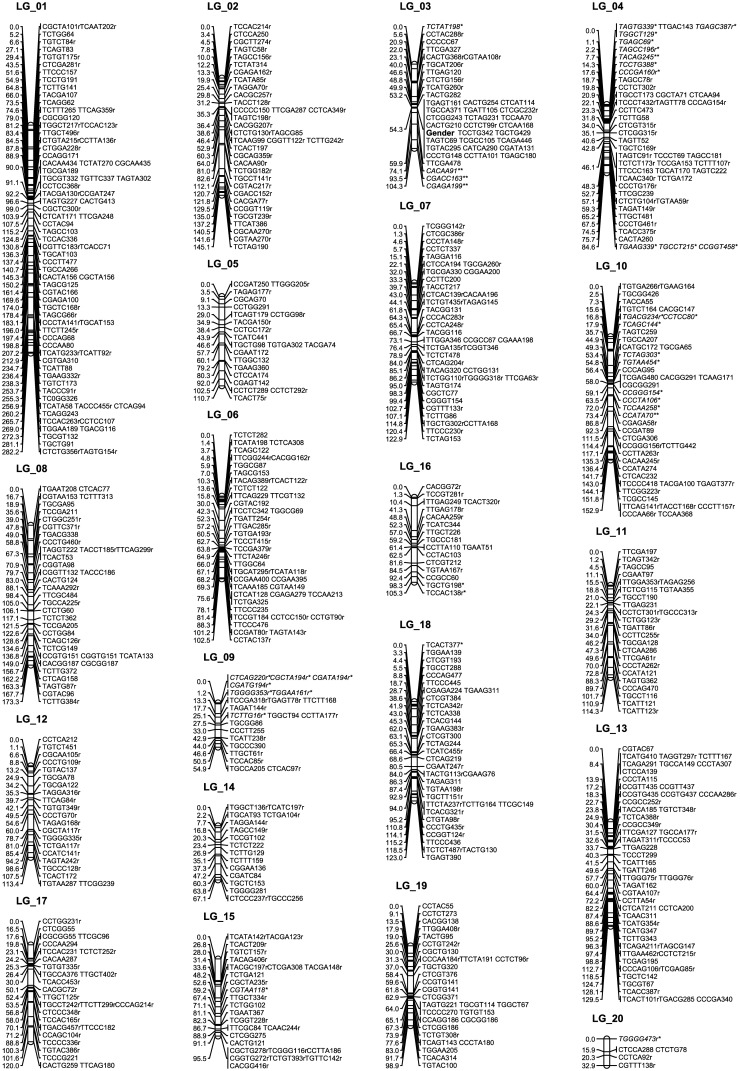
AFLP genetic map for the maternal parent. ^a^ The gender locus is shown in bold font in LG_03. ^b^ Markers with a “*” or a “**” indicate segregation distortion at a significance level of 0.05 or 0.01, respectively.

AFLP genotyping was conducted following the protocol as described by Yin et al. [40], and PCR products were detected on an ABI 3730 sequencer (Applied Biosystems, CA, USA). Clearly segregated amplicons in range of 50–500 bp were extracted and recorded by using GeneMapper Software (Version 4.0, Applied Biosystems, CA, USA). Mendelian segregation of markers was assessed with the chi-square test. In addition to the 1:1 segregated markers, those with significant departure from Mendelian segregation (P≤0.05) were also included to build the genetic map. The intercross markers (heterozygous in both parents) were excluded from map construction. Mapmaker (Version 3.0) [41] was employed to perform map construction following the two-way pseudo-testcross strategy [42], and the mapping procedure was conducted as described by Yin et al. [40]. The map charts were drawn with MapChart (Version 2.1) [43]. Segregation distorted markers were indicated with a “*” at significance level of P≤0.05, or a “**” at significance level of P≤0.01 (Fig 1).

### Scoring the gender phenotype and mapping the gender locus

For each of the mapping progeny, two ramets were planted for the field trial. Normally, one-year-old *S*. *suchowensis* can achieve sexual maturity,, and flowers in spring through March to April. Gender of each ramet was scored in 2013, 2014, and 2015. To position the gender locus, the gender phenotype data were duplicated both in coupling and repulsion phases. Then, both phases of the gender marker were separately mapped as 1:1 segregating markers to detect linkage with AFLPs on the maternal and paternal maps.

### Marker distribution analysis

Whether markers were overabundant or dispersed among linkage groups was assessed as described by Remington et al. [44]. Under the assumption of an even distribution of markers, the expected number of markers, λ_*i*_, was calculated as:*λ*_*i*_ = *mL*_*i*_ /∑*L*_*i*_, where *m* was the total number of mapped markers, and *L*_*i*_ was the observed map length of linkage group *i*. The probabilities that a linkage group contained more or fewer markers than the expected number λ_*i*_ were evaluated by using a two-tailed cumulative Poisson calculator. Subsequently, clustering and dispersion of markers within each linkage group was evaluated by opening sliding-windows along each linkage group, following the method described by Yin et al. [40]. In this analysis, a new window was opened if genetic distances among at least three continuous markers were smaller than the average.

### Sex chromosome identification

In willows, sex chromosomes were found to be associated with chromosome XV [11, 26]. To identify the linkage group that corresponds to the willow sex chromosome in this study, simple sequence repeat (SSR) primer pairs were designed with sequences of scaffolds in the vicinity of the gender locus on chromosome XV [11]. SSR genotyping was conducted as that described in Zhou et al. [45]. The PCR products were analyzed on an ABI 3730 sequencer (Applied Biosystem). The segregated SSRs were selected and used for genotyping the mapping population. SSR genotypes were recorded as co-dominant markers, and integrated into the established linkage groups with Mapmaker (Version 3.0) [41]. Based on the mapping result, the linkage group that corresponds to the willow sex chromosome was identified.

## Results

### Genetic map construction

An initial of 448 AFLP primer combinations were screened with the two mapping parents and six progeny. Based on the gel profile, 204 primer combinations that generated highly clear and polymorphic bands were selected and used for genotyping the mapping population. In total, 1,943 segregating loci were yielded. Among these,, 650 were maternally informative, 622 were paternally informative, and 671 were intercross markers. Because intercross markers might lead to biased estimates of recombination rates [46], they were excluded from map construction. At the LOD threshold of 4.0, the maternal testcross markers were assigned into 20 linkage groups and mapped into 457 marker bins. The built map spanned a total genetic length of 2333.3 cM, with linkage group sizes ranging from 32.9 to 282.2 cM (Fig 1). Alternatively, in the paternal map, the 622 testcross markers were assigned into 21 linkage groups and mapped into 441 marker bins at the LOD threshold of 4.0. The established map spanned a total genetic distance of 2260.0 cM, with linkage group sizes ranging from 45.6 to 248.6 cM (Figure A in S1 File).

On the maternal map, segregation of 36 markers was significantly distorted from the 1:1 segregation ratio (Fig 1), which accounted for 5.5% of the maternal testcross markers. Significant segregation distorted marker clusters were observed on linkage groups 03, 04, 09, and 10. For the paternal map, 29 segregation-distorted markers were detected, which accounted for 4.7% of the paternal testcross markers, and significant segregation distorted marker clusters mainly occurred on linkage groups 07, 13, and 18.

### Mapping the gender locus

For the 92 mapping progeny, gender phenotypes were observed on different ramets for three continuous years. Among these, 47 were female and 45 were male. Segregation of gender did not deviate from the 1:1 Mendelian segregation ratio. Mapped as a 1:1 segregating morphological marker, the gender locus was positioned in the centromeric region of LG_03 on the maternal map (Fig 1), but it was not mapped on the paternal map (Figure A in S1 File).

### Marker distribution analysis

Analyzing marker distribution among linkage groups revealed that, on the maternal map, AFLP markers were overabundant on linkage groups 04, 06, and 13, whereas markers were sparse on linkage groups 12, 16, and 20. The most abundant and dispersed distribution of AFLP markers occurred on LG_04 and LG_16, respectively (Table 1). In the paternal map, overabundances of AFLP markers were observed on linkage groups 04, 10, 15, and 21, and marker distribution was sparse on linkage groups 01, 17, and 20; additionally, the most abundant and dispersed distributions of AFLP markers was observed on LG_21 and LG_01, respectively (Table A in S1 File).

**Table 1:** Analysis of marker distribution among linkage groups on the maternal map.

Linkage group	The observed map length (cM)	The expected map length (cM)	The expected number of AFLPs	The observed number of AFLPs	Poisson two-tailed P-value ^a^
LG_01	282.2	289.63	75.32	77	0.4383
LG_02	145.1	153.64	39.95	35	0.2447
LG_03	104.3	109.94	28.59	38	0.0527
LG_04	84.6	88.36	22.98	46	0.0000**^+^
LG_05	110.7	121.77	31.67	21	0.0296
LG_06	102.5	107.63	27.99	41	0.0124*^+^
LG_07	122.9	129.20	33.60	40	0.1543
LG_08	173.3	182.93	47.57	37	0.0679
LG_09	54.9	60.39	15.70	21	0.1159
LG_10	152.9	160.18	41.65	43	0.4379
LG_11	114.3	123.83	32.20	25	0.1160
LG_12	113.4	124.74	32.44	21	0.0219*^−^
LG_13	129.5	134.68	35.02	51	0.0066*^+^
LG_14	67.1	76.05	19.78	16	0.2359
LG_15	95.5	103.14	26.82	26	0.4881
LG_16	105.3	118.46	30.81	17	0.0050**^−^
LG_17	120	129.23	33.61	27	0.1450
LG_18	123	130.45	33.92	34	0.5176
LG_19	98.9	105.96	27.56	29	0.4166
LG_20	32.9	49.35	12.83	5	0.0120*^−^
Total	2333.3	2499.55	650.00	650	

^a^ “*” indicates a significance level of 0.05,

“**” indicates a significance level of 0.01.

“^+^” following the “*” or “**” indicates that markers are overabundant on the corresponding linkage group.

“^−^” following the “*” or “**” indicates that markers are sparse on the corresponding linkage group.

Because this is a two-tailed test, a P-value of 0.025 corresponds to a significance level of 0.05.

Overabundance or dispersion of AFLPs was observed within each linkage group on both the maternal and paternal map. In total, 96 sliding windows were opened on the maternal map. Among these, 26 windows were detected to be overabundant with AFLPs, and markers were sparse in six windows. There were 32.8% of the AFLPs mapped in the marker-clustered regions, but these regions only represented 6.8% of the total maternal map distance. It is noteworthy that AFLPs were extremely condensed in the sliding window that contained the gender locus on LG_03. This window spanned a genetic distance of 1.1 cM, which accounted for 1.1% of the total length of LG_03. However, 23 markers (which accounted for 60.5% of the total markers in this linkage group) were mapped onto the corresponding region. In the maternal map, the AFLP-dispersed regions covered a genetic distance of 340.9 cM (14.6% of the total length), but contained only 7.7% of the total mapped markers (Table B in S1 File). In the paternal map, a total of 84 sliding windows were opened. Among these, overabundance of AFLPs occurred in 29 of the sliding windows, and dispersion of AFLPs was observed in nine of the sliding windows. Marker clustered regions were found to contain 41.3% of the mapped AFLPs, but only spanned 10.1% of the total paternal map distance. On the paternal map, the AFLP-dispersed regions spanned a genetic distance of 425.7 cM (18.8% of the total length), and were comprosed of only 10.3% of the total paternally mapped AFLP markers (Table C in S1 File). Besides the marker clustered and dispersed windows, there were 59.4% and 48.4% sliding windows with numbers of AFLPs that were not significantly deviated from the expectations on the maternal and on the paternal maps, respectively.

### Sex chromosome identification

**Fig 2:**
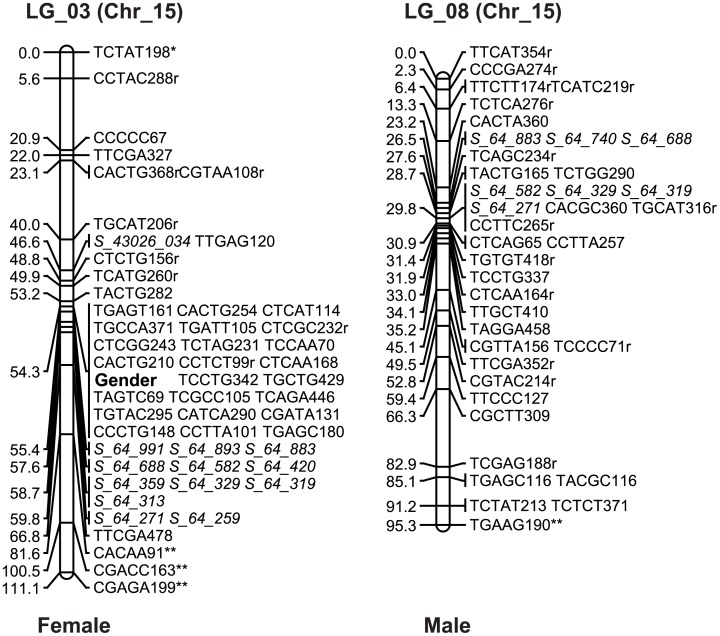
Linkage group associated with the willow sex chromosome in the maternal and paternal parents. ^a^ The gender locus is shown in bold in LG_03 of the maternal parent. ^b^ SSRs are displayed in italics. ^c^ Markers with a “*” or a “**” indicate segregation distortion at a significance level of 0.05 or 0.01, respectively.

Pucholt et al. [26] reported that turnover of sex chromosome was associated with chromosome XV in *S*. *viminalis* L., and the same finding was reported in Hou et al. [11]. According to the genome sequence of *S*. *suchowensis* [11, 47], we designed 50 SSR primers based on sequence of chromosome XV, and 14 of these primers generated segregated markers in the mapping pedigree (Table D in S1 File). Among these, seven SSRs (S43026_034, S_64_991, S_64_893, S_64_420, S_64_359, S_64_313, S_64_259) were maternally informative, one SSR (S_64_740) was paternal informative, and six SSRs (S_64_883, S_64_688, S_64_582, S_64_329, S_64_319, S_64_271) were fully informative. Mapping of the segregated SSRs revealed that LG_03 was associated with willow chromosome XV (Fig 2). Thus, LG_03 of the maternal map corresponds to chromosome XV, which is the sex chromosome of willow. On the paternal map, the segregated SSRs were mapped on LG_08. Although this linkage group did not contain the gender locus, when aligned with the fully informative SSRs, the homologous regions between the female’s LG_03 and the male’s LG_08 could be identified (Fig 2). In the corresponding region of the paternal map, an overabundance of AFLP markers was observed, but the gender locus was unmappable in this region (Table C in S1 File).

## Discussion

Linkage mapping is a fundamental platform for genetically dissecting the location and organization of genes associated with traits of interest. In this study, high-density linkage maps were constructed for the mapping parents of an *S*. *suchowensis* full-sib F_1_ cross. In the established maps, genetic length of LG_01 was much larger than that of any other linkage groups in both the female and male (Fig 1 and Figure A in S1 File). A gigantic linkage group was also observed in mapping studies of different poplar species [10, 28, 40]. Therefore, this gigantic linkage group should not be an artifact of mapping. In early cytological studies, a gigantic chromosome was commonly observed in *Populus* [48–52]. Willows and poplars originated from a common ancestor [47, 52], and the two lineages shared high collinearity between their genomes [37]. Thus, willows might also possess a gigantic chromosome as in poplars. Genome sequencing projects revealed that the ancestor of willows and poplars was a paleotetrapolyploid [47, 53], which resulted from a whole-genome duplication event, called “salicoid” duplication. After the salicoid duplication, a radical re-organization of the genome occurred. The modern *Salicaceae* chromosomes were found to arise from extensive Robertsonian rearrangements (i.e., fusion of two centromeres into one, or the fission of one centromere into two) [53]. We propose that the gigantic chromosome in *Salicaceae* species might be due to fusion of centromeres of ancestral chromosomes.

In *Salicaceae* species, the base chromosome number is 19, and most members in *Salicaceae* family exist in the diploid form [54, 55]. The first genetic map that contained 19 linkage groups in poplar was established by Yin et al. [40]. Berlin et al. [38] built a genetic map for *S*. *viminalis* L., for which linkage groups number equivalent to the chromosome number. Very recently, Hou et al. [11] reported a complete genetic map that was composed of 19 linkage groups in *S*. *suchowensis*. In this study, we obtained 20 and 21 linkage groups for the maternal and paternal map, respectively. The exceeded number of linkage groups may be due to the uneven distribution of AFLP restriction sites. Additionally, non-captured gaps on the linkage map may be related to the existence of recombination hot spots in particular regions of the willow genome. Additionally, genotyping errors could also obstruct the linkage [34]. Although the established genetic maps did not achieve complete coverage of the willow genome, the gender locus region was covered by the maternal and paternal maps. It is notable that an overabundance of AFLP markers was observed in the gender region on both maps. Clusters of AFLPs might merely be due to an overabundance of AFLP recognition sites; however, it could also be an indication of recombination suppression. Recombination suppression was found to be an important cause of heterogeneities in marker density along linkage maps [56, 57]. In the hypothesis for the evolution of dioecy, organisms are proposed to have required development of local mechanisms to prevent recombination [58, 59]. Recombination suppression at the sex determination locus was regarded as the key event that promoted the evolution of sex chromosomes [12, 58, 60, 61]. Severe recombination suppression has been observed in the vicinity of the sex determination locus in different dioecious plants [10, 14]. In papaya, the recombination suppression region, which contains the gender locus, was confirmed to be located in centromere of the sex chromosome [62]. Thus far, all mapping studies in willows [11, 26], together with this work, positioned the gender locus in the middle of a linkage group, a region of which corresponded to willow sex chromosome. Since the chromosomes of *Salicaceae* are typically metacentric [48, 63], we suspect that the gender locus of willow is also located in the centromeric region; however, direct situ-hybridization evidence from cytological studies is needed to test this idea.

In willows, sex was consistently found to occur through a ZW system, in which the female was the heterogametic gender [11, 26]. By contrast, both female and male heterogamety has been reported in members of *Populus* [10, 28, 30]. It is possible that both ZZ/ZW and XX/XY sex-determination systems could be present in some members of the family *Salicaceae* [23]. Besides the sex determination system, the mapping position of the gender locus was consistent in different willow species. However, the location of the gender locus also varied among different poplars, with a peritelomeric localization in members of the *Aigeiros* [28] and *Tacamahaca* subgenera [10], and a centromeric localization in subgenera of *Leuce* [27, 29, 30, 64]. Sex chromosomes have arisen several times in flowering plant evolution [31], and sex chromosomes are evolutionarily young in some plants compared with most mammals [65, 66]. Recent studies revealed that different autosomes evolved into sex chromosomes in the sister genera of *Salix* and *Populus*, and the appearance of sex chromosomes occurred after the divergence of these two lineages [11, 26]. The sex-determination systems are still at an early evolutionary stage and could be very diverse in different members of *Salicaceae*. Thus, *Salicaceae* species provide a desirable system to study the genetics and evolution of sex chromosomes.

Dioecy in *Salicaceae* species is strongly genetically controlled, although there are very rarely noted examples of gender reversion and hermaphroditic plants [10, 27–30, 64, 67]. Diverse genetic bases of sex determination, including sex chromosomes, simple Mendelian genes, quantitative genes, environment, and genotype-by-environment interactions, have been proposed in *Salicaceae* [10, 23, 26, 68]. However, all mapping studies pointed to single-locus sex determination in both willows and poplars [10, 11, 25–29, 30, 64, 67, 69]. Although gender was confirmed be controlled by a single locus, this locus might encompass several genes that underlie gender determination. Recombination suppression would render all genes within this region tightly linked, and they would segregate as one locus. If more than one gene determines gender, recombination would impair sexual differentiation. To maintain separate sexes, the genes that determine maleness or femaleness would have to be closely linked on the alternate chromatids of sex chromosomes, and this region would have to develop local mechanisms to prevent recombination [58, 59]. Contrary to this traditional perspective, a very recent study demonstrated that a single gene could cause dioecy [70].

In this study, we found that a single locus governed sex determination in willow, and the female was the heterogamic gender, which was consistent with the findings of Pucholt et al. [26] and Hou et al. [11]. Although the exact sex determination gene has not been cloned, this study provides desirable information to learn the genetic basis and chromosomal signatures associated with sex determination in willow. Family of *Salicaceae* is a well-studied woody plant system at the molecular level. The genomes of several species in this family had been sequenced [47, 53, 71], and numerous transcriptome sequences are available in the public databases [67, 72]; together with mapping studies, it will be possible to clone the exact sex determinant(s) of species in family of *Salicaceae* in the near future.

## Supporting Information

S1 File **Table A. Analysis of marker distribution among linkage groups on the paternal map.** ^a^ “*” indicates a significance level of 0.05, and “**” indicates a significance level of 0.01. “+” following the “*” or “**” indicates that markers are overabundant on the corresponding linkage group. “-” following the “*” or “**” indicates that markers are sparse on the corresponding linkage group. Because this is a two-tailed test, a P-value of 0.025 corresponds to a significance level of 0.05. **Table B. Analysis of marker distribution within each linkage group of the maternal map.** ^a^ “*” indicates a significance level of 0.05, and “**” indicates a significance level of 0.01. “+” following the “*” or “**” indicates that markers are overabundant on the corresponding linkage group. “-” following the “*” or “**” indicates that markers are sparse on the corresponding linkage group. Because this is a two-tailed test, a P-value of 0.025 corresponds to a significance level of 0.05. **Table C. Analysis of marker distribution within each linkage group of the paternal map.** ^a^ “*” indicates a significance level of 0.05, and “**” indicates a significance level of 0.01. “+” following the “*” or “**” indicates that markers are overabundant on the corresponding linkage group. “-” following the “*” or “**” indicates that markers are sparse on the corresponding linkage group. Because this is a two-tailed test, a P-value of 0.025 corresponds to a significance level of 0.05. **Table D. Segregated AFLP markers developed from sequence scaffolds mapped on willow’s chromosome XV. Figure A. AFLP genetic map for the paternal parent.** ^a^ Markers with “*” or “**” indicate segregation distortion at a significance level of 0.05 or 0.01, respectively.
(PDF)
