# Supplementary material for: Confirmation of Single-Locus Sex Determination and Female Heterogamety in Willow Based on Linkage Analysis
Source: PLoS One. 2016 Feb 1;11(2):e0147671. doi: 10.1371/journal.pone.0147671 (PMC4734660; doi:10.1371/journal.pone.0147671)
Supplement: S1 File — Table A. Analysis of marker distribution among linkage groups on the paternal map. a “*” indicates a significance level of 0.05, and “**” indicates a significance level of 0.01. “+” following the “*” or “**” indicates that markers are overabundant on the corresponding linkage group. “-” following the “*” or “**” indicates that markers are sparse on the corresponding linkage group. Because this is a two-tailed test, a P-value of 0.025 corresponds to a significance level of 0.05. Table B. Analysis of marker distribution within each linkage group of the maternal map. a “*” indicates a significance level of 0.05, and “**” indicates a significance level of 0.01. “+” following the “*” or “**” indicates that markers are overabundant on the corresponding linkage group. “-” following the “*” or “**” indicates that markers are sparse on the corresponding linkage group. Because this is a two-tailed test, a P-value of 0.025 corresponds to a significance level of 0.05. Table C. Analysis of marker distribution within each linkage group of the paternal map. a “*” indicates a significance level of 0.05, and “**” indicates a significance level of 0.01. “+” following the “*” or “**” indicates that markers are overabundant on the corresponding linkage group. “-” following the “*” or “**” indicates that markers are sparse on the corresponding linkage group. Because this is a two-tailed test, a P-value of 0.025 corresponds to a significance level of 0.05. Table D. Segregated AFLP markers developed from sequence scaffolds mapped on willow’s chromosome XV. Figure A. AFLP genetic map for the paternal parent. a Markers with “*” or “**” indicate segregation distortion at a significance level of 0.05 or 0.01, respectively. (PDF) [file pone.0147671.s001.pdf]

Table S1 Analysis of marker distribution among linkage groups on the paternal map

Linkage group	The observed map length (cM)	The expected map length (cM)	The expected number of AFLPs	The observed number of AFLPs	Poisson two-tailed P-value
LG_01	248.6	260.44	66.61	43	0.0013**[-]
LG_02	156.6	164.63	42.11	40	0.4117
LG_03	125.3	131.41	33.6	42	0.0901
LG_04	96.8	101.64	26.00	41	0.0039**[+]
LG_05	107.8	116.09	29.69	27	0.3534
LG_06	107.4	114.56	29.30	31	0.4010
LG_07	122.5	132.30	33.84	26	0.1000
LG_08	93.0	99.20	25.37	31	0.1542
LG_09	45.6	57.00	14.58	9	0.0847
LG_10	95.8	101.12	25.86	37	0.0228*[+]
LG_11	104.9	112.13	28.68	30	0.4272
LG_12	75.2	82.36	21.07	22	0.4480
LG_13	114.6	125.02	31.97	23	0.0615
LG_14	48.5	55.96	14.31	14	0.5373
LG_15	151.8	157.42	40.26	55	0.0157*[+]
LG_16	87.4	96.14	24.59	21	0.2735
LG_17	153.1	164.04	41.95	29	0.0225*[-]
LG_18	104.1	112.78	28.84	25	0.2731
LG_19	62.2	71.77	18.36	14	0.1857
LG_20	59.6	72.84	18.63	10	0.0221*[-]
LG_21	99.2	103.09	26.37	52	0.0000**[+]
Total	2260.0	2431.95	622.00	622	

[a] "*" indicates a significance level of 0.05, and "**" indicates a significance level of 0.01. "+" following the "*" or "**" indicates that markers are overabundant on the corresponding linkage group. "-" following the "*" or "**" indicates that markers are sparse on the corresponding linkage group. Because this is a two-tailed test, a P-value of 0.025 corresponds to a significance level of 0.05.

Table S2 Analysis of marker distribution within each linkage group of the maternal map

Linkage group	Windows	Map interval (cM)	The expected number of AFLPs	The observed number of AFLPs	Poisson two-tailed P-value
LG_01	1	[0, 79]	21.56	15	0.0908
	2	[79, 92.2]	3.60	17	0.0000**$^{+}$
	3	[92.2, 253.7]	44.07	32	0.0358
	4	[253.7, 272.3]	5.08	10	0.0346
	5	[272.3, 282.2]	2.70	3	0.5067
LG_02	1	[0, 13.3]	3.21	7	0.0451
	2	[13.3, 29.8]	3.98	3	0.4374
	3	[29.8, 38.6]	2.12	8	0.0016**$^{+}$
	4	[38.6, 135]	23.25	13	0.0154*$^{-}$
	5	[135, 145.1]	2.44	4	0.2289
LG_03	1	[0, 20.9]	7.61	3	0.0548
	2	[20.9, 23.1]	0.80	3	0.0476
	3	[23.1, 53.2]	10.97	5	0.0383
	4	[53.2, 54.3]	0.40	23	0.0000**$^{+}$
	5	[54.3, 104.3]	18.22	4	0.0001**$^{-}$
LG_04	1	[0, 2.2]	1.20	6	0.0015**$^{+}$
	2	[2.2, 17.6]	8.37	3	0.0329
	3	[17.6, 23.3]	3.10	9	0.0047**$^{+}$
	4	[23.3, 46.1]	12.40	6	0.0367
	5	[46.1, 46.1]	0.00	10	0.0000**$^{+}$
	6	[46.1, 84.6]	20.93	12	0.0253
LG_05	1	[0, 3.5]	0.66	3	0.0299
	2	[3.5, 34.9]	5.96	5	0.4527
	3	[34.9, 46.6]	2.22	5	0.0746
	4	[46.6, 110.7]	12.16	8	0.1448
LG_06	1	[0, 7]	2.80	8	0.0081*$^{+}$
	2	[7, 60.5]	21.40	11	0.0105*$^{-}$
	3	[60.5, 78.1]	7.04	15	0.0060*$^{+}$
	4	[78.1, 102.5]	9.76	7	0.2426
LG_07	1	[0, 43]	14.00	12	0.3589
	2	[43, 44.1]	0.36	3	0.0059*$^{+}$
	3	[44.1, 66.7]	7.36	4	0.1430
	4	[66.7, 84]	5.63	7	0.3346
	5	[84, 86.2]	0.72	5	0.0009**$^{+}$
	6	[86.2, 122.9]	11.94	9	0.2473
LG_08	1	[0, 67.3]	14.37	11	0.2302
	2	[67.3, 70.9]	0.77	4	0.0079*$^{+}$
	3	[70.9, 134.6]	13.60	12	0.3989
	4	[134.6, 136.8]	0.47	3	0.0122*$^{+}$

	5	[136.8, 173.3]	7.79	7	0.4822
LG_09	1	[0, 1.2]	0.46	6	0.0000**$^{+}$
	2	[1.2, 25.1]	9.14	5	0.1073
	3	[25.1, 27.5]	0.92	3	0.0659
	4	[27.5, 54.9]	10.48	7	0.1800
LG_10	1	[0, 2.5]	0.70	3	0.0345
	2	[2.5, 15.6]	3.68	2	0.2881
	3	[15.6, 17.9]	0.65	4	0.0044**$^{+}$
	4	[17.9, 53.4]	9.98	5	0.0677
	5	[53.4, 59.1]	1.60	7	0.0013**$^{+}$
	6	[59.1, 111.5]	14.74	6	0.0090*$^{-}$
	7	[111.5, 117.1]	1.57	3	0.2102
	8	[117.1, 141.7]	6.92	3	0.0861
	9	[141.7, 152.9]	3.15	10	0.0016**$^{+}$
LG_11	1	[0, 4.5]	0.98	3	0.0774
	2	[4.5, 15.5]	2.41	2	0.5682
	3	[15.5, 24.3]	1.92	7	0.0037**$^{+}$
	4	[24.3, 114.3]	19.69	13	0.0752
LG_12	1	[0, 13.2]	2.44	5	0.1015
	2	[13.2, 34.2]	3.89	2	0.2548
	3	[34.2, 42.1]	1.46	3	0.1819
	4	[42.1, 113.4]	13.20	11	0.3328
LG_13	1	[0, 8.4]	3.31	8	0.0200*$^{+}$
	2	[8.4, 17.2]	3.47	2	0.3272
	3	[17.2, 33.7]	6.50	14	0.0071*$^{+}$
	4	[33.7, 95.2]	24.22	14	0.0179*$^{-}$
	5	[95.2, 98.8]	1.42	5	0.0150*$^{+}$
	6	[98.8, 128.1]	11.54	5	0.0271
	7	[128.1, 129.5]	0.55	3	0.0186*$^{+}$
LG_14	1	[0, 2.2]	0.52	4	0.0021**$^{+}$
	2	[2.2, 16.8]	3.48	2	0.3243
	3	[16.8, 26.9]	2.41	3	0.4325
	4	[26.9, 60.3]	7.96	4	0.1017
	5	[60.3, 67.1]	1.62	3	0.2222
LG_15	1	[0, 26.8]	7.30	3	0.0676
	2	[26.8, 33.6]	1.85	5	0.0402
	3	[33.6, 86.7]	14.46	8	0.0495
	4	[86.7, 95.5]	2.40	10	0.0002**$^{+}$
LG_16	1	[0, 48.8]	7.88	6	0.3284
	2	[48.8, 62.5]	2.21	6	0.0255
	3	[62.5, 105.3]	6.91	5	0.3124
LG_17	1	[0, 16.5]	3.71	2	0.2833
	2	[16.5, 30]	3.04	10	0.0012**$^{+}$
	3	[30, 58]	6.30	7	0.4418

	4	[58, 120]	13.95	8	0.0636
LG_18	1	[0, 8.8]	2.43	5	0.1000
	2	[8.8, 38.6]	8.24	4	0.0868
	3	[38.6, 45.3]	1.85	3	0.2833
	4	[45.3, 68.6]	6.44	5	0.3777
	5	[68.6, 95.2]	7.35	11	0.1254
	6	[95.2, 123]	7.68	6	0.3534
LG_19	1	[0, 30]	8.80	7	0.3482
	2	[30, 31.3]	0.38	3	0.0070*[+]
	3	[31.3, 58.4]	7.95	2	0.0143*[-]
	4	[58.4, 67.3]	2.61	11	0.0001**[+]
	5	[67.3, 98.9]	9.27	6	0.1836
LG_20	1	[0, 32.9]	5.00	5	0.6160
Total	96		650.00	650	

[a] "*" indicates a significance level of 0.05, and "**" indicates a significance level of 0.01. "+" following the "*" or "**" indicates that markers are overabundant on the corresponding linkage group. "-" following the "*" or "**" indicates that markers are sparse on the corresponding linkage group. Because this is a two-tailed test, a P-value of 0.025 corresponds to a significance level of 0.05.

Table S3 Analysis of marker distribution within each linkage group of the paternal map

Linkage group	Windows	Map interval (cM)	The expected number of AFLPs	The observed number of AFLPs	Poisson two-tailed P-value
LG_01	1	[0, 70.1]	12.13	12	0.5617
	2	[70.1, 80.1]	1.73	4	0.0977
	3	[80.1, 145.1]	11.24	5	0.0324
	4	[145.1, 148.8]	0.64	7	0.0000**$^{+}$
	5	[148.8, 215.1]	11.47	9	0.2920
	6	[215.1, 226.6]	1.99	3	0.3204
	7	[226.6, 248.6]	3.81	3	0.4724
LG_02	1	[0, 63.4]	16.19	11	0.1177
	2	[63.4, 67]	0.92	3	0.0661
	3	[67, 79.7]	3.24	9	0.0062*$^{+}$
	4	[79.7, 155.5]	19.36	12	0.0518
	5	[155.5, 156.6]	0.28	5	0.0000**$^{+}$
LG_03	1	[0, 42.6]	14.28	12	0.3315
	2	[42.6, 53.7]	3.72	14	0.0000**$^{+}$
	3	[53.7, 125.3]	24.00	16	0.0563
LG_04	1	[0, 6.6]	2.80	9	0.0024**$^{+}$
	2	[6.6, 54]	20.08	9	0.0048**$^{-}$
	3	[54, 67.1]	5.55	20	0.0000**$^{+}$
	4	[67.1, 96.8]	12.58	3	0.0015**$^{-}$
LG_05	1	[0, 5.5]	1.38	5	0.0134*$^{+}$
	2	[5.5, 51.8]	11.60	6	0.0572
	3	[51.8, 57.3]	1.38	6	0.0030**$^{+}$
	4	[57.3, 92.4]	8.79	2	0.0074*$^{-}$
	5	[92.4, 96.8]	1.10	4	0.0259
	6	[96.8, 107.8]	2.76	4	0.2981
LG_06	1	[0, 31.4]	9.06	4	0.0529
	2	[31.4, 58.8]	7.91	19	0.0006**$^{+}$
	3	[58.8, 107.4]	14.03	8	0.0612
LG_07	1	[0, 12.5]	2.65	7	0.0189*$^{+}$
	2	[12.5, 116.9]	22.16	15	0.0724
	3	[116.9, 122.5]	1.19	4	0.0328
LG_08	1	[0, 23.2]	7.73	5	0.2169
	2	[23.2, 32.9]	3.23	13	0.0000**$^{+}$
	3	[32.9, 93]	20.03	13	0.0652
LG_09	1	[0, 45.6]	9.00	9	0.5874
LG_10	1	[0, 7.7]	2.97	3	0.5709
	2	[7.7, 8.8]	0.42	3	0.0093*$^{+}$
	3	[8.8, 17.5]	3.36	8	0.0217*$^{+}$
	4	[17.5, 42.9]	9.81	4	0.0331
	5	[42.9, 48.4]	2.12	6	0.0215*$^{+}$

	6	[48.4, 95.8]	18.31	13	0.1276
LG_11	1	[0, 2.2]	0.63	8	0.0000**$^{+}$
	2	[2.2, 63.2]	17.45	9	0.0207*$^{-}$
	3	[63.2, 66.7]	1.00	3	0.0805
	4	[66.7, 94.7]	8.01	3	0.0422
	5	[94.7, 104.9]	2.92	7	0.0295
LG_12	1	[0, 3.7]	1.08	4	0.0245*$^{+}$
	2	[3.7, 18]	4.18	7	0.1307
	3	[18, 75.2]	16.73	11	0.0946
LG_13	1	[0, 2.3]	0.46	4	0.0013**$^{+}$
	2	[2.3, 26.9]	4.94	3	0.2740
	3	[26.9, 36.9]	2.01	7	0.0046**$^{+}$
	4	[36.9, 114.6]	15.59	9	0.0528
LG_14	1	[0, 40.4]	11.66	8	0.1785
	2	[40.4, 48.5]	2.34	6	0.0321
LG_15	1	[0, 16.7]	5.94	14	0.0033**$^{+}$
	2	[16.7, 76.2]	21.17	11	0.0118*$^{-}$
	3	[76.2, 80.8]	1.64	8	0.0003**$^{+}$
	4	[80.8, 99.4]	6.62	5	0.3523
	5	[99.4, 100.5]	0.39	3	0.0075*$^{+}$
	6	[100.5, 135.1]	12.31	5	0.0167*$^{-}$
	7	[135.1, 138.4]	1.17	3	0.1149
	8	[138.4, 151.8]	4.77	5	0.5177
LG_16	1	[0, 30.2]	7.60	4	0.1248
	2	[30.2, 42.2]	3.02	10	0.0012**$^{+}$
	3	[42.2, 87.4]	11.38	8	0.2002
LG_17	1	[0, 48.9]	9.26	7	0.2941
	2	[48.9, 58.9]	1.89	9	0.0002**$^{+}$
	3	[58.9, 110.3]	9.74	5	0.0778
	4	[110.3, 120.7]	1.97	3	0.3152
	5	[120.7, 148.6]	5.28	2	0.1026
	6	[148.6, 153.1]	0.85	3	0.0552
LG_18	1	[0, 15.6]	3.75	13	0.0001**$^{+}$
	2	[15.6, 104.1]	21.25	12	0.0218*$^{-}$
LG_19	1	[0, 4.4]	0.99	8	0.0000**$^{+}$
	2	[4.4, 62.2]	13.01	6	0.0257
LG_20	1	[0, 56.7]	9.51	7	0.2673
	2	[56.7, 59.6]	0.49	3	0.0134*$^{+}$
LG_21	1	[0, 38.3]	20.08	7	0.0007**$^{-}$
	2	[38.3, 51.2]	6.76	18	0.0002**$^{+}$
	3	[51.2, 82.8]	16.56	6	0.0028**$^{-}$
	4	[82.8, 85]	1.15	9	0.0000**$^{+}$
	5	[85, 96]	5.77	4	0.3176
	6	[96, 99.2]	1.68	8	0.0004**$^{+}$
Total	84		622.00	622	

[a] “*” indicates a significance level of 0.05, and “**” indicates a significance level of 0.01. “+” follow the “*” or “**” indicates that markers are overabundant on the corresponding linkage group. “-” follow the “*” or “**” indicates that markers are sparse on the corresponding linkage group. Because this is a two-tailed test, a P-value of 0.025 corresponds to a significance level of 0.05

Table S4 Segregated AFLP markers developed from sequence scaffolds mapped on willow's chromosome XV

Primer Name	Primer Left Sequence	Primer Right Sequence	Scaffold Name	Start Position (bp)	End Position (bp)	Informativeness
S_43026_034	TTTCCTTTTCTTCTGACA	TATCTTGACCAGGAGTGG	scaffold43026	340	353	Maternal
S_64_259	GATAACAACAAGATGGGTC	AACTGTAAATGAGCGGTA	scaffold64	259348	259380	Maternal
S_64_271	CACAAATCTTATTGGAAAAC	TTACTACTGATGCTGTTC	scaffold64	271110	271148	Fully
S_64_313	GGACGAATCCAACCTGAC	CCTGCTTGCCTGAATCT	scaffold64	313514	313534	Maternal
S_64_319	CATCGTGCCCAGTAAGGA	ACATAGGAAGCGGGTGGT	scaffold64	319671	319694	Fully
S_64_329	AGAGTCCGCAAGGAAGGG	GCCAAAGCCACCAGATAA	scaffold64	329783	329802	Fully
S_64_359	AAATCAATACCCGTGGAA	TTCTTGCTGCTCAATCTG	scaffold64	359829	359850	Maternal
S_64_420	AAAGAATCCTACCAAACA	GAGCTGTCATATTCCCTA	scaffold64	420327	420338	Maternal
S_64_582	GTCTGAACCCTCATCTAT	CTGGAATCCATAATACAC	scaffold64	582928	582945	Fully
S_64_688	GCAAAAGCCAAAAGGAGA	AACCAGCAGAGGAAAGTG	scaffold64	688403	688420	Fully
S_64_740	AGAGCAAAGCACATTTCA	ATACATCTACTGCCACCC	scaffold64	740338	740363	Paternal
S_64_883	AAAAGGTTATTTGATACACG	CATTCCACTAAAGACATTGA	scaffold64	883414	883433	Fully
S_64_893	TAGTTGGGCCACGGAAAC	ATGGAGCAAATGGTAATAGGAG	scaffold64	893780	893817	Maternal
S_64_991	TAGTGGTTCAACCTTCTT	AACATTGGTTTCTTGATT	scaffold64	991141	991162	Maternal

Fig. S1 AFLP genetic map for the paternal parent

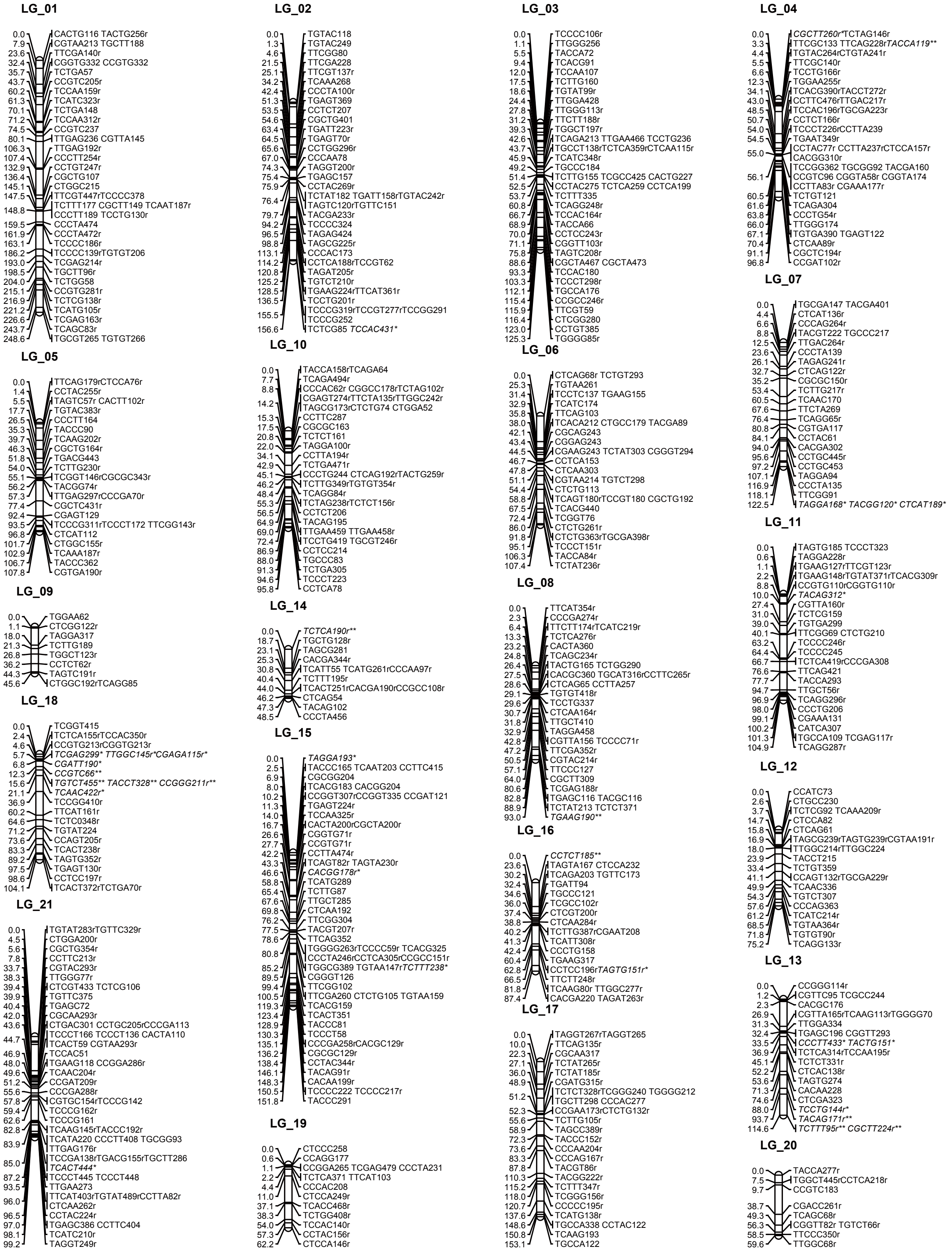

[a] Markers with "*" or "**" indicate segregation distortion at a significance level of 0.05 or 0.01, respectively
